# Fine Mapping of Leaf Trichome Density Revealed a 747-kb Region on Chromosome 1 in Cold-Hardy Hybrid Wine Grape Populations

**DOI:** 10.3389/fpls.2021.587640

**Published:** 2021-03-03

**Authors:** Lu Yin, Avinash Karn, Lance Cadle-Davidson, Cheng Zou, Anna Underhill, Paul Atkins, Erin Treiber, Daniel Voytas, Matthew Clark

**Affiliations:** ^1^Department of Horticultural Science, University of Minnesota, Twin Cities, MN, United States; ^2^Institute of Biotechnology, Bioinformatics Facility, Cornell University, Ithaca, NY, United States; ^3^United States Department of Agriculture, Agricultural Research Service, Grape Genetics Research Unit, Geneva, NY, United States; ^4^Department of Genetics, Cell Biology, and Development, University of Minnesota, Twin Cities, MN, United States

**Keywords:** QTL mapping, fine mapping, trichome density, candidate gene, hybrid grape, phylloxera resistance

## Abstract

Segregation for leaf trichome density was observed in a cold-hardy hybrid grape population GE1025 (*N* = ∼125, MN1264 × MN1246) that was previously used to detect a quantitative trait locus (QTL) underlying foliar phylloxera resistance on chromosome 14. Our hypothesis was that high trichome density was associated with resistance to phylloxera. Existing literature found trichome density QTL on chromosomes 1 and 15 using a hybrid grape population of “Horizon” × Illinois 547-1 and suggested a few candidate genes. To validate the reported QTL and our hypothesis, interval mapping was conducted in GE1025 with previous genotyping-by-sequencing (GBS) single nucleotide polymorphism (SNP) genotype data and phenotypic scores collected using a 0–6 trichome density scale at several leaf positions. Evaluations were done on replicated forced dormant cuttings in 2 years and on field-grown leaves in 1 year. There was no strong relationship between trichome density and phylloxera resistance except for a Pearson’s correlation (r) of about -0.2 between a few trichome density traits and phylloxera severity traits at 2 and 3 weeks after infestation. Two genetic regions were repeatedly detected for multiple trichome density traits: from 10 to 20.7 Mbp (∼10 Mbp) on chromosome 1 for ribbon and simple density traits and from 2.4 to 8.9 Mbp on chromosome 10 for ribbon density traits, explaining 12.1–48.2 and 12.6–27.5% of phenotypic variation, respectively. To fine map, we genotyped a larger population, GE1783 (*N* = ∼1,023, MN1264 × MN1246), with conserved rhAmpSeq haplotype markers across multiple *Vitis* species and phenotyped 233 selected potential recombinants. Evaluations were conducted on field-grown leaves in a single year. The QTL for ribbon trichome density on adaxial vein and adaxial leaf and simple density on abaxial vein was fine mapped to 12.63–13.38 Mbp (747 kb) on chromosome 1. We found variations of MN1264 and MN1246 at candidate genes NAC transcription factor 29, EF-hand protein, and MYB140 in this region and three other surrounding candidate genes proposed previously. Even though no strong relationship between foliar phylloxera resistance and trichome density was found, this study validated and fine mapped a major QTL for trichome density using a cold-hardy hybrid grape population and shed light on a few candidate genes that have implications for different breeding programs.

## Introduction

**FIGURE 1:**
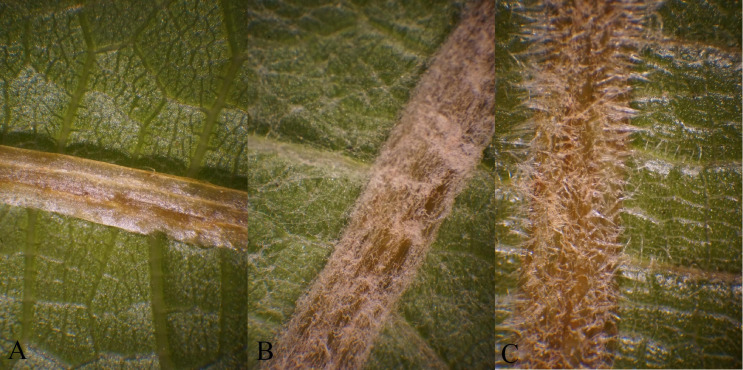
Trichome types observed on abaxial leaf blade and veins in a cold-hardy hybrid grape population, GE1025, as an example for **(A)** no trichome, **(B)** ribbon trichome, and **(C)** simple trichome.

Trichomes are epidermis cell projections on surfaces of different organs of plants (Schmidt, 2014) and in the *Vitis* genus are a distinctive feature for taxa identification (ampelography) (Cheng et al., 2013; Gerrath et al., 2015; Ma et al., 2016). Importantly, trichomes are well-known to play a role in plant defense (Schmidt, 2014). In numerous plant systems, there is a negative correlation between trichome density and small herbivorous arthropods feeding, oviposition responses, and larval development (Levin, 1973). There are also examples of trichomes positively impacting the performance of herbivorous arthropods (Barba et al., 2019). While glandular trichomes can secrete secondary metabolites that make plant defense more complicated, the current study focuses on non-glandular trichomes. Non-glandular trichomes are categorized by Ma et al. (2016) as ribbon and simple types (Figure 1).

Trichomes are one morphological feature that plants use to combat insect pests while the most widely accepted resistance mechanisms include antixenosis, antibiosis, and tolerance (Painter, 1951). Antixenosis refers to the plants having certain characteristics, including morphological and chemical, that are non-preferable to the pest (Pedigo and Rice, 2009). With morphological antixenosis, plants have structures that physically impede the normal behaviors of the pest, as is the case for non-glandular leaf trichomes (Pedigo and Rice, 2009). Morphological antixenosis provides a first line of defense that is not easily overcome by the pest as opposed to most chemical-based resistance (Pedigo and Rice, 2009). Thus, trichomes are one of the potential durable resistance targets for plant breeders.

Trichomes have been a target for breeding programs in a range of crop species. Trichomes have been studied in several dicot species including *Brassica* spp., field bean *Phaseolus* cultivars, and soybean (*Glycine max*) (Levin, 1973; Agren and Schemske, 1992). Glabrous mutants were found to be unfavorable to whitefly in cotton (*Gossypium arboreum*) and favorable to larvae of a white butterfly and a cabbage fly in *Arabidopsis halleri* (Sato and Kudoh, 2015; Grover et al., 2016). Previous work on the glabrous cotton mutant suggests that a single recessive gene was responsible for absence of trichomes (Baljinder et al., 2011). In *Arabidopsis* spp., a rich pool of literature is available that has studied gene regulatory networks involved in trichome patterning. These include major genes GLABRA1 (GL1), myb domain protein 23 (MYB23), GLABRA3 (GL3), its homolog, and TRANSPARENT TESTA GLABRA1 (TTG1) (Grebe, 2012). In *Vitis*, Barba et al. (2019) found trichome density quantitative trait locus (QTL) on chromosomes 1 and 15 using a hybrid grape population of “Horizon” × Illinois 547-1 and suggested a few candidate genes such as GL1, GLABROUS INFLORESCENCE STEMS2 (GIS2), zinc finger protein 8 (ZFP8), and REPRESSOR of GA1-3 (RGA1-3). However, the QTL for trichome density on chromosomes 1 and 15 found by Barba et al. (2019) ranged from 3 to 8 Mbp, a relatively wide region to identify the candidate gene.

We observed segregation for leaf trichome density in a cold-hardy hybrid wine grape F_1_ population GE1025 (*N* = ∼125, MN1264 × MN1246; Teh et al., 2017) that was previously used to detect a QTL underlying foliar phylloxera resistance on chromosome 14 (Clark et al., 2018). Phylloxera, *Daktulosphaira vitifoliae* (Fitch), is especially a problem on foliage of cold-hardy hybrid wine grapes, which dominate *Vitis* species grown in the Midwest (Yin et al., 2019). We hypothesized that high trichome density was associated with resistance to foliar phylloxera. MN1264 and MN1246 are two advanced University of Minnesota (UMN) grape breeding selections with 6 *Vitis* in their pedigree. The phylloxera-resistant parent, MN1264, showed more trichomes than the susceptible parent, MN1246. Anecdotally, dense ribbon trichomes in the *Vitis labrusca* hybrid “Edelweiss” were noted alongside low foliar phylloxera infestation.

This study aimed to validate the previously reported trichome QTL and its relationship to foliar phylloxera resistance using the established population GE1025 (Experiment 1) to fine map the QTL using a larger F_1_ population GE1783 (*N* = ∼1,023, MN1264 × MN1246) that was used to fine map phylloxera resistance QTL (unpublished) (Experiment 2) and to sequence targeted trichome candidate genes in the parents of the populations (Experiment 3) for a better understanding of candidate gene functions and making more informed breeding decisions.

## Materials and Methods

### QTL Mapping in GE1025 Population (Experiment 1)

#### Plant Materials

In the winters of 2018 and 2019, three replications of dormant, hardwood stem cuttings were collected from each field-grown GE1025 genotype as top-wire high-cordon trained vines at the UMN Horticultural Research Center (44°52’08.1”N, 93°38’17.3”W). In 2018, cuttings were collected on February 14 and stored at 4°C until March 14 to ensure that chilling requirements were met. In 2019, cuttings were collected on December 20, 2018 and stored at 4°C until January 25. Dormant, two-node cuttings were made and moved to water (2018) or perlite (2019) to induce budbreak. Cuttings were maintained at the Plant Growth Facilities at the UMN Saint Paul campus at an average temperature of 22°C and a day length of 16 h until budbreak (∼1 month).

In the summer of 2019, three replications of the first mature leaf (observed as the oldest leaf that is still tender but is fully expanded) were collected from each field-grown GE1025 genotype (hereafter referred to as “2019 field”). Leaves were kept in plastic bags and maintained on ice until trichome scoring.

#### Trichome Scoring

**FIGURE 2:**
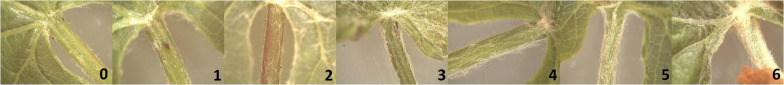
A representative visual scale to rate ribbon trichome density on adaxial petiole on first mature leaf samples.

**FIGURE 3:**
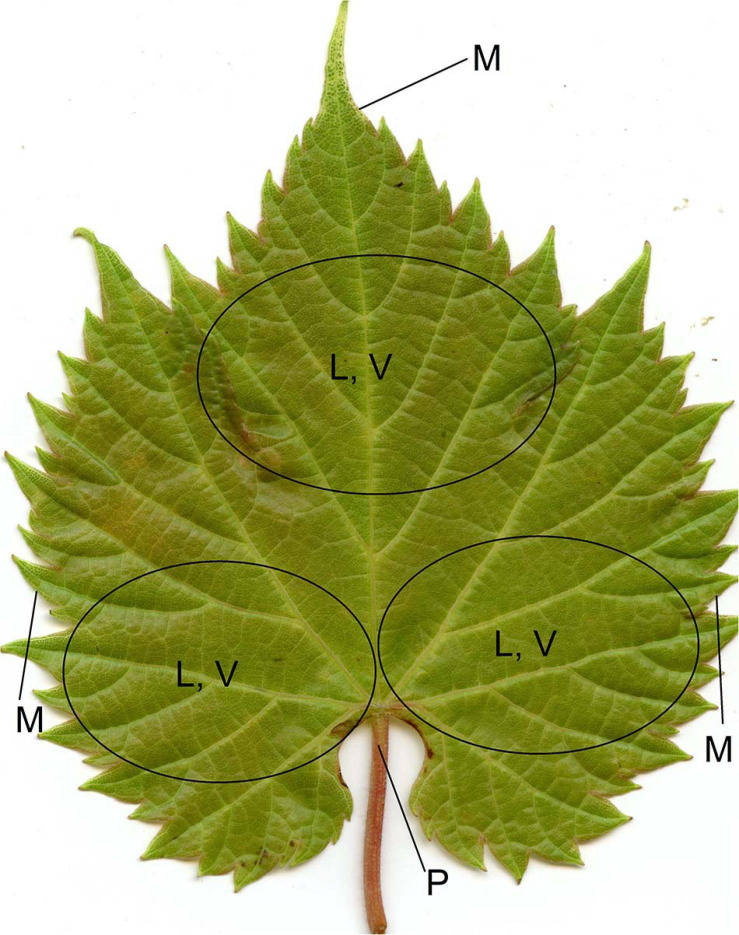
Leaf positions where trichomes were scored: leaf blade (L), vein (V), petiole (P), and margin (M; only on the adaxial side) on both sides of the leaf. A comprehensive score was assigned to each of L, V, P, and M by evaluating these multiple areas.

Ribbon and simple types of trichomes (Figure 1) were scored for their density using a 0–6 rating scale (Figure 2) under 22.5 × magnification using an Omano^®^ stereo dissecting microscope. In 2018 and 2019, the first mature/last tender leaf from the first node of each cutting was recorded. In 2019 field, the collected leaves were kept at 4°C and scored within 4 days. When the first mature leaf was too small or the first node died, measurements were taken on the next available leaf or the lower node. The density of trichomes was recorded on various leaf positions on both sides of the leaf including major leaf vein, leaf blade between veins (hereafter referred to as “leaf”), petiole, and leaf margin (recorded only on the adaxial side) (Figure 3). Several density traits not recorded in 2018 were added in 2019; only trichomes on leaf and vein were scored in 2019 field samples (Supplementary Table 1).

#### Phenotypic Analysis

Pearson correlations were calculated between trichome density traits across all three experiments and foliar phylloxera traits measured previously by Clark et al. (2018) using *rcorr* function in “Hmisc” package and visualized by the “corrplot” package in R version 3.6.0 (Wei and Simko, 2017; Harrell, 2019; R Core Team, 2019). Narrow-sense heritabilities (h^2^) for trichome density were calculated as ∑_g_^2^/((g - 1)/r × σ^2^ + ∑_g_^2^), where ∑_g_^2^ is the variance due to genotypes (fixed), g is the number of genotypes, r is the number of replications, and σ^2^ is the variance due to error (Bernardo, 2010).

Two-sided *t*-tests with unequal variances of foliar phylloxera traits (Clark et al., 2018) were performed on trichome presence/absence; presence was defined as occurring in at least one replication, and absence was defined as lacking in all three replications of each GE1025 genotype for each leaf position.

Analysis of variance (ANOVA) was conducted on trichome density through fitting a linear model of genotype as a fixed effect and replication as a random effect. Error normality of the linear model and variance homogeneity among GE1025 genotypes were examined using *plot.lm* function and *leveneTest* function in “car” package in R (Fox and Weisberg, 2019). Natural log and square root transformations were performed on density traits that did not have residual normality. If a transformation improved the error variance to normal or nearly normal, that transformation was used. If a trichome density trait had a non-normal error distribution or unequal variances, no ANOVA results were reported.

#### Phenotypic Analysis of Parental Checks

To confirm our hypothesis that the parent MN1264 (MN1069 × “Seyval Blanc”) had more trichomes than the other parent MN1246 (MN1200 × “Frontenac”), phenotypic scores of the parents and grandparents were compared and with that of “Edelweiss” (hairy).

Specifically, three replications of greenhouse-grown MN1264, MN1246, “Edelweiss,” and “Frontenac” were scored for trichome density in 2018, 2019, and 2019 field in the QTL mapping experiment. Up to five replications of greenhouse-grown (except MN1246 was field-grown) MN1264, MN1246, and grandparents were scored in the fine mapping experiment (described below). Mean scores of ribbon and simple types on leaf and vein were compared using *TukeyHSD* function in R after fitting a linear model of effects of genotype by experiment (2018, 2019, 2019 field, and fine mapping experiment) and replication. The model was examined by *anova* function in R with *F*-value of genotype effect manually corrected by MS_genotype_/MS_genotype × experiment_ (MS: mean square), and the residual normality was checked using the *plot.lm* function. For traits with normally distributed errors and a significant genotype effect, Tukey’s HSD test was conducted, and trait means were visualized using *ggplot2* (Wickham, 2016).

#### Genotyping

Genotyping-by-sequencing (GBS) single nucleotide polymorphism (SNP) data used for GE1025 population were published previously by Teh et al. (2017). GBS data were generated following *Ape*KI restriction digestion as described by Elshire et al. (2011), using 384 barcodes for library preparation (Hyma et al., 2015) with Illumina single-end HiSeq 2000 sequencing (San Diego, CA, United States). Maternal and paternal linkage maps were constructed in JoinMap 4.1 using 1,649 and 1,375 high-quality biallelic SNP markers, respectively, based on PN40024 12X v1 reference genome (Adam-Blondon et al., 2004; Cipriani et al., 2011; Adam-Blondon, 2014; Canaguier et al., 2014; Teh et al., 2017).

#### QTL Analysis

Interval mapping using *scanone* function of *R/qtl* was used to conduct QTL analysis on trichome density traits (Broman, 2009). For trichome density traits with approximately normal residuals, a multiple imputation method was performed with 128 imputations and a step size of 1. For trichome density traits with non-normal residual distributions, the Haley–Knott (HK) method was performed with a step size of 1. The 5% logarithm of odds (LOD) threshold for significant QTL was determined for both methods by 1,000 permutations. Effect plots were examined for each marker at the peak of a QTL, and the high trichome density allele and low trichome density allele were recorded.

### Fine Mapping in GE1783 Population (Experiment 2)

#### Plant Materials

Based on preliminary QTL results of experiment 1, 183 recombinant genotypes from population GE1783 within 11.7–20.7 Mbp on chromosome 1 and 3–9 Mbp on chromosome 10 were selected for phenotyping. Because the QTL on chromosome 1 (10–18.09 Mbp) for simple density on adaxial vein was slightly shifted from the QTL for other traits, a slightly different but overlapping set of 233 genotypes were selected for evaluation. Due to the young vine age of GE1783 plants, available leaf materials for replication were variable. One to five replications of the first mature/last tender leaf of these field-grown genotypes were collected in three separate days in the first week of October 2019 at the UMN Horticultural Research Center. Those leaves were kept in plastic bags and maintained on ice until digital images were acquired through scanning.

#### Trichome Scoring

Leaves were scanned using the Epson^®^ Perfection V550 Photo Scanner (Long Beach, CA, United States) Office mode within 24 h of collection. Replications of the same genotype were scanned together under 1,200 dpi, flipped to the other side of the leaves, scanned, and cropped around the leaves. Each scanned image was viewed in Windows Photo Viewer, zoomed in, and scored using the same density scale (Figure 2).

#### Genotyping

rhAmpSeq genotyping was conducted on ∼1,023 GE1783 genotypes (Zou et al., 2020). Briefly, 2,000 amplicon markers targeting 250 bp regions evenly distributed across the *Vitis* core genome (average marker distance = 200 kb) based on PN40024 12X v2 reference sequence were pair-end sequenced on Illumina NextSeq 500 (Canaguier et al., 2017; Zou et al., 2020). Because multiple SNPs, insertions, and/or deletions can occur in each 250 bp amplicon, these markers represent multi-allelic haplotypes, returning up to four alleles per locus in this F_1_ population of two heterozygous parents (Supplementary Figure 10 in Zou et al., 2020). A sex-averaged consensus genetic map was constructed from 1,387 quality-controlled haplotype markers in LepMap3 (Rastas, 2017) as previously described (Zou et al., 2020).

#### Haplotyping Analysis

To fine map the trichome QTL regions that were repeatedly detected in experiment 1, rhAmpSeq data of recombinant genotypes at each locus of interest were associated with phenotypes collected in experiment 2. Genotypes were organized into graphical haplotype classes with high (H) or low trichome density (L) haplotypes spanning the QTL region. Based on results of experiment 1, the genotypic codings “11” and “21” were H haplotypes (having allele “1” at the second position/in the mother), and “12” and “22” were L haplotypes (having allele “2” in the mother). The genetic region where the presence (or loss) of the H (or L) haplotype was associated with the high (or low) density phenotype was determined as the fine mapped region. ANOVA was conducted fitting haplotype class, number of genotypes within each haplotype class, and replication effects using *lm* and *anova* functions in R with the *F*-value of haplotype effect manually corrected by MS_haplotype_/MS_haplotype/genotypes_. For ribbon trichome density, an additional rater effect was included. For traits with non-normal residuals, natural log or square root transformations were performed, and their residuals were re-examined for improvement in normality. ANOVA was not conducted if the assumption of residual normality was not met. For traits with a significant haplotype class effect, means of H and L haplotypes were separated using Tukey’s HSD at the fine mapped region using the *glht* function in *multcomp* package in R (Hothorn et al., 2008).

### Sequencing of Candidate Trichome Genes in the Parents (Experiment 3)

#### Shotgun Sequencing at the Fine Mapped Region

The parents, MN1264 and MN1246, were whole-genome pair-end sequenced using Illumina HiSeq 2500 with about 20-fold read coverage (Zou et al., 2020). Using bwa mem (Li, 2013; RRID:SCR_010910), fastq sequences of each parent were aligned to PN40024 12X v2 reference sequence with v3 annotation (Canaguier et al., 2017). SNPs were annotated using the SnpEff program (Cingolani et al., 2012). Coverage of MN1264 and MN1246 at the fine mapped region was generated by the Gviz package (Hahne and Ivanek, 2016). Reads over 100× coverage were ignored because they represent repetitive regions on the chromosome.

#### Nanopore Sequencing at Three Other Genes on Chromosome 1

Although outside of our fine mapped region, three candidate genes from Barba et al. (2019) were examined providing possible information for this trait in this genetic background. MN1264 and MN1246 were sequenced using long-range PCR and nanopore sequencing based on PN40024 12X v1 reference genome (Adam-Blondon et al., 2004; Cipriani et al., 2011; Adam-Blondon, 2014; Canaguier et al., 2014) for consistency with that used by Barba et al. (2019).

For long-range PCR, primers were designed with sample-specific barcodes targeting these candidate genes at 8.1 (GL1/MYB23), 9.7 (GIS2/ZFP8), and 19.2 Mbp (RGA/RGA-LIKE 1) based on CRIBI v2.1 annotation (Supplementary Table 2) following the guidelines according to Chauhan (2019) using Primer3Plus (Untergasser et al., 2012) and IDT PrimerQuest^®^ (Coralville, Iowa). DNA of MN1264 and MN1246 was extracted using Qiagen DNeasy^®^ 96 Plant Kit (Venlo, the Netherlands) and stored at -40°C. For two of the regions (9.7 and 19.2 Mbp), the rapid PCR protocol was performed as follows: 1 μl of ∼50 ng/μl DNA, 2 μl of the PrimeSTAR^®^ GXL DNA polymerase (Takara Bio Inc., Japan), 1 μl of 10 μM forward primer, 1 μl of 10 μM reverse primer, 4 μl of dNTP mixture, and 10 μl of 5X PrimeSTAR^®^ buffer to a final volume of 50 μl at 98°C for 10 s and 68°C for 4 min of 30 cycles. For the third region (8.1 Mbp), the standard PCR protocol was performed with the same reagents except for 1 μl of DNA polymerase. PCR was performed at 98°C for 10 s, 60°C for 15 s, and 68°C for 2.5 min of 30 cycles. To confirm the presence and approximate size of the PCR products, samples were visualized on 1.5% agarose gel with 1 μg/ml ethidium bromide after running under 80 V for 1.5 h. PCR products were cleaned using QIAquick^®^ PCR Purification Kit (Venlo, the Netherlands) and stored at -40°C.

Nanopore sequencing using the Flongle adapter was conducted on pooled PCR products across samples at candidate regions (Oxford Nanopore Technologies, United Kingdom; RRID:SCR_017985). To prepare sequencing libraries, the ligation sequencing kit SQK-LSK109 was used (Oxford Nanopore Technologies, United Kingdom). Samples were demultiplexed in minibar (Krehenwinkel et al., 2019), and basecalling was done by Guppy version 3.3.3 + fa743a6. To compare variation between MN1264 and MN1246 at these three regions, each of their sequences was aligned to the PN40024 12X v1 reference genome using minimap2 (Li, 2018; RRID:SCR_018550) and samtools (Li et al., 2009).

For each sample at each region, the bam file of the nanopore sequencing data was imported into Geneious^®^ (Auckland, New Zealand; RRID:SCR_010519) to generate a consensus sequence at 25% threshold with default settings. For samples displaying a heterozygous insertion/deletion, the bam reads were manually separated into one bam file with the insertion/deletion and one bam file without the insertion/deletion, and two consensus sequences were generated to represent the heterozygous nature of that variation for that individual. For visualization, the consensus fasta files of MN1264 and MN1246 were aligned to the PN40024 12X v1 reference using SnapGene^®^ (San Diego, CA, United States; RRID:SCR_015052).

#### Comparison of Trichome Density on “Pinot” Mutants at GAI1

To examine the effect of one of these candidate genes on trichome density, we scored trichomes on scanned images of three *Vitis vinifera* varieties “Pinot Noir,” “Pinot Meunier,” and “Pinot Pixie” on leaf and vein using the 0–6 scale as descried above. “Pinot Meunier” has a point mutation in the L1 epidermis layer in one allele of GAI1 (GAI1/gai1; see “Results” section for more information) that is expected to have dense trichomes otherwise it is genetically identical to “Pinot Noir” (Cousins, 2012). “Pinot Pixie” is a semi-dwarf variety that was regenerated from the L1 epidermis layer of “Pinot Meunier” and was expected to inherit the dense trichome phenotype (GAI1/gai1) from “Pinot Meunier” (Skene and Barlass, 1983; Boss and Thomas, 2002; Cousins, 2012). At each sample position, ANOVA was conducted fitting a linear model of genotype, environment (Minnesota or New York), and replication effects. Residual normality and variance equality were also checked. *TukeyHSD* was conducted to separate means of these genotypes, and results were visualized using *ggplot2* (Wickham, 2016).

## Results

### Consistent Phenotypic Scores Across Years/Experiments

Phenotypic correlations (r) ranged from 0.46 to 0.79 high between 2019 cuttings and 2019 field evaluations for ribbon and from 0.22 to 0.75 for simple trichome density in GE1025 (Figure 5). The checks, including the parents, were consistent across all evaluations (2018, 2019, 2019 field, and fine mapping experiment). The hirsute *V. labrusca* based variety “Edelweiss” had higher ribbon density than “Frontenac” except for abaxial vein in 2019, whereas “Frontenac” had higher simple density on vein and edge than “Edelweiss” (Supplementary Figure 2; edge data not shown). Phenotypic trichome scores are deposited at https://doi.org/10.13020/mqze-pw21.

In general, MN1264 had numerically higher trichome density than MN1246 for ribbon trichome density on abaxial vein, adaxial leaf, and adaxial vein and simple trichome density on abaxial vein in all evaluations (Supplementary Figure 2). The exception was for simple trichome density on adaxial vein where MN1246 had numerically higher value than MN1264 (Supplementary Figure 2). The sources of high ribbon trichome density of MN1264 likely came from MN1069 on adaxial leaf and vein. The source of high simple trichome density of MN1246 on adaxial vein likely came from “Frontenac.” Normality of residuals and equal variances were not met for ribbon and simple density on abaxial leaf. The equal variances assumption was also not met for simple density on adaxial leaf.

### QTL Mapping Revealed Two Regions for Multiple Trichome Density Traits

**FIGURE 4:**
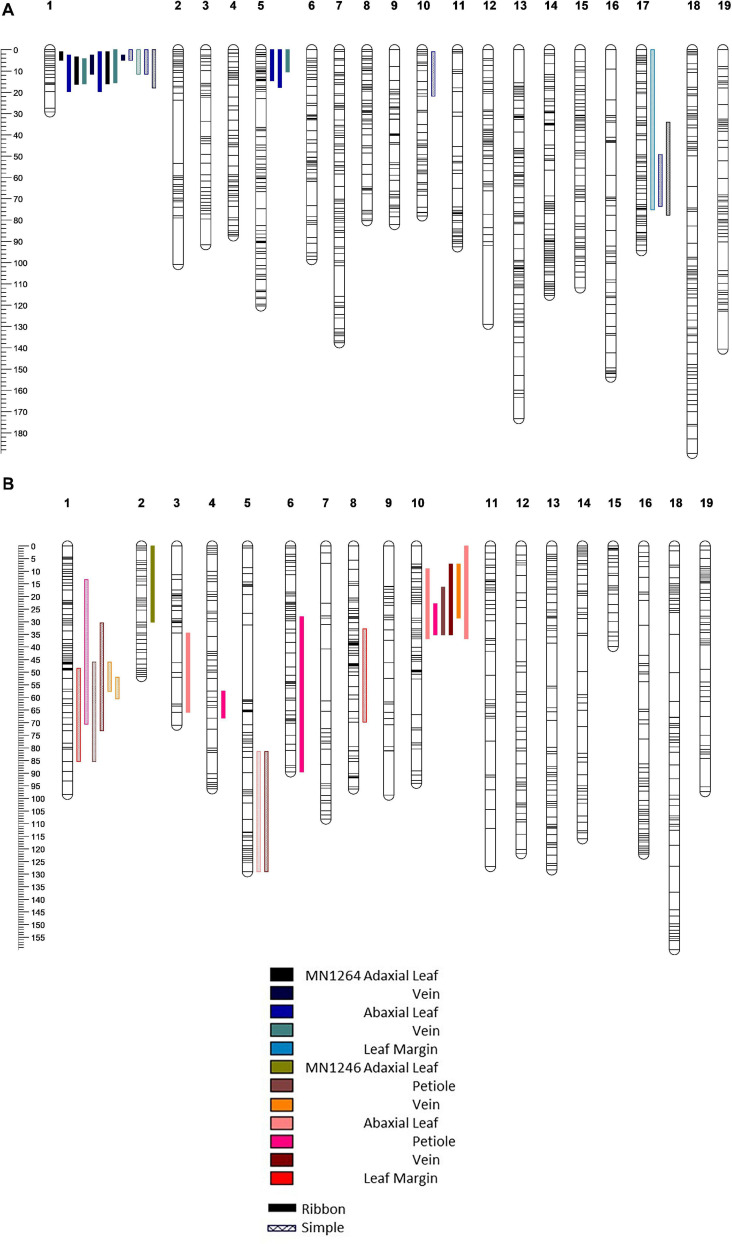
Quantitative trait loci detected on the **(A)** MN1264 map and **(B)** MN1246 map of a hybrid grape population GE1025 of trichome density of ribbon and simple types on adaxial and abaxial leaf surfaces and various leaf positions using scanone function of interval mapping (R/qtl). Multiple bars of the same color and pattern suggest that the trait has been detected in multiple experiments (2018, 2019, or 2019 field). QTL bars represent 95% Bayesian intervals.

Narrow-sense heritability of trichome density at each sample position ranged from 64 to 95% in GE1025. In total, we detected a single QTL for eight trichome density traits and two or three QTL for five traits (Figure 4 and Tables 1, 2). We observed only one interaction between the QTL on chromosomes 1 and 5 for abaxial leaf ribbon trichome density in 2019 and 2019 field (data not shown).

**TABLE 1:** Ribbon trichome density QTL found in a cold-hardy hybrid grape population, GE1025, with GBS SNP markers using multiple imputation (imp) and Haley–Knott (HK) method of scanone Rqtl with 95% Bayesian intervals.

	Side of leaf	Trait^a^	Chr	Marker	Position (cM)	Interval (cM)	Interval (Mbp)	LOD	%Phenotypic variation
MN1264 map	Adaxial	Leaf 18	1	S1_15222119	4.2	0.9–5	12–17.2	7.3	27.2
		Leaf 19	1	S1_18704100	6.6	3.3–16.3	13–21	7.2	26.8
		Leaf 19F	1	S1_16785806	4.2	0.9–16	11.9–21	8.1	28.4
		Vein 19	1	S1_18231605	6.6	2.5–11.5	12.9–19.7	15.6	47.8
		Vein 19F	1	S1_16785806	4.2	2.5–5	12.9–17.2	16.6	48.2
	Abaxial	√leaf 19	1	S1_17146981	5.0	2.5–19.7	12.9–22.1	3.9	14.4
			5	S5_1424484	0.0	0–14.6	1.4–3.6	3.4	13.0
		Leaf 19F^b^	1	S1_19075517	7.4	0.9–19.7	12–22.1	3.9	13.8
			5	S5_2388377	1.3	0–17.8	1.4–4.2	3.2	11.6
		Vein 19	1	S1_19817756	9.8	4.2–16	15.2–21	5.5	21.8
		Vein 19F	1	S1_19817756	9.8	0–15.5	11.7–20.7	4.3	13.8
			5	S5_2191983	3.2	0–10.5	1.4–2.4	4.5	14.3
MN1246 map	Adaxial	Leaf 18	2	S2_5064468	21.4	0–30.2	1.3–NA^c^	4.5	18.4
		Vein 19	10	S10_3523645	21.2	7.2–28.6	NA^c^–4.9	3.3	14.8
		log_e_ petiole 19	10	S0_15860662	22.9	16.3–35.2	2.2–6.6	7.3	27.5
	Abaxial	√leaf 19	3	S3_6543591	46.2	34.5–65.9	4.5–12.3	3.1	11.5
			10	S10_5314224	29.5	9–36.8	1.2–8.9	3.2	12.6
		Leaf 19F^b^	10	S0_15860662	22.9	0–36.8	2.5–8.9	3.0	13.8
		Vein 19	10	S10_3523645	21.2	7.2–35.2	NA^c^–6.6	4.5	18.7
		Petiole 19	4	S4_17966014	59.9	57.5–68.2	17.8–20.5	4.7	10.8
			6	S6_20918375	81.0	28–89.5	7.9–22.2	3.1	9.0
			10	S10_5599086	32.8	22.9–35.2	NA^c^–6.6	6.8	19.6

*^a^Trichome density measured at that position on the leaf in 2018 (18), 2019 (19), or 2019 field (19F); some traits were transformed by square root (√) or natural log (log_e_). ^b^Residuals non-normally distributed even after transformation. ^c^Markers with a physical position on other or unknown chromosomes based on the current reference assembly.*

**TABLE 2:** Simple trichome density QTL found in a cold-hardy hybrid grape population, GE1025, with GBS SNP markers using multiple imputation (imp) and Haley–Knott (HK) method of scanone Rqtl with 95% Bayesian intervals.

	Side of leaf	Trait^a^	Chr	Marker	Position (cM)	Interval (cM)	Interval (Mbp)	LOD	% Phenotypic variation
MN1264 map	Adaxial	Leaf 19F^b^	17	S17_8448103	67.4	34–77.7	5.0–10	4.3	15.2
		Vein 19F^b^	1	S1_12888845	2.5	0–18	11.7–NA^e^	4.4	15.3
	Abaxial	Leaf 19^b^	1	S1_15222119	4.2	0–11.5	11.7–19.7	4.9	18.1
		Leaf 19F	1	S1_12888845	2.5	0–5	11.7–17.2	8.8	12.1
			10	S10_1472990	9.9	0.9–21.8	0.2–3.6	3.5	ns^cd^
			17	S17_6831362	54.8	49.2–73.6	6.3–9	3.9	ns^c^
		Vein 19F	1	S1_11717050	0.0	0–11.5	11.7–19.7	9.1	31.2
	Leaf margin 18		17	S17_6004065	42.3	0–75.2	0.2–9.7	3.6	15.3
								
MN1246	Adaxial	Vein 19	1	S1_16785854	56.7	46–57.6	10–17.6	4.1	17.3
		Vein 19F^b^	1	S1_12871638	54.0	52–60.5	12.8–18.1	7.9	26.1
		log_e_ petiole 19	1	S1_16785854	56.7	46–85.4	10–21	3.2	14.1
	Abaxial	Leaf 19F	5	S5_25478657	125.5	81.4–129.1	17.6–NA^e^	3.2	13.6
		Vein 19	1	S1_12871638	52.5	30.5–73.2	6.2–19.6	3.0	13.3
		Vein 19F	5	S5_22919310	116.6	81.4–129.1	17.6–NA^e^	4.0	16.3
		√petiole 19	1	S1_16785854	56.7	13.3–70.6	3.7–19.6	3.3	14.7
	Leaf margin 18		1	S1_11908918	49.2	48.4–85.4	11.2–21	3.9	16.3
								
	Leaf margin 19		8	S8_14646580	49.9	32.8–69.8	9.8–17.2	3.5	15.0

*^a^Trichome density measured at that position on the leaf in 2018 (18), 2019 (19), or 2019 field (19F); some traits were transformed by square root (√) or natural log (log_e_). ^b^Non-normally distributed residuals fitting a linear model of genotype effect and replication effect (even after transformations) where HK method, instead of imp, was used. ^c^ns: non-significant QTL when included with the other two QTL. ^d^Significant at 0.1. ^e^Markers with a physical position on other or unknown chromosomes based on the current reference assembly.*

For genetic regions on chromosomes 1, 5, 10, and 17, we detected coincident QTL for multiple trichome density traits. Due to the number of traits measured, we will discuss ribbon and simple trichome separately.

#### Ribbon Trichome Density

Four ribbon trichome density traits were mapped to 11.7–20.7 Mbp on chromosome 1 (hereafter referred to as the “chromosome 1 QTL”), and five traits were mapped to 2.4–8.9 Mbp on chromosome 10 (hereafter referred to as the “chromosome 10 QTL”) according to the PN40024 12X v1 reference (Figure 4 and Table 1). The chromosome 1 QTL was found on the MN1264 map and explained 13.8–48.2% of phenotypic variation for ribbon trichome density. The chromosome 10 QTL was found on the MN1246 map and explained 12.6–27.5% variation for ribbon density (Table 1). The chromosome 1 QTL for adaxial leaf and vein positions was repeatedly detected in two experiments (Table 1).

#### Simple Trichome Density

Six simple trichome density traits were mapped to the chromosome 1 QTL region (10–20.7 Mbp for adaxial vein), which explained 12.1–31.2% variation on the MN1264 map and 13.3–26.1% variation on the MN1246 map (Figure 4 and Table 2). QTL for abaxial leaf, abaxial vein, and adaxial vein positions has been detected in two or more experiments (Table 2).

### A Weak Negative Relationship Between Foliar Phylloxera Severity and Trichome Density

**FIGURE 5:**
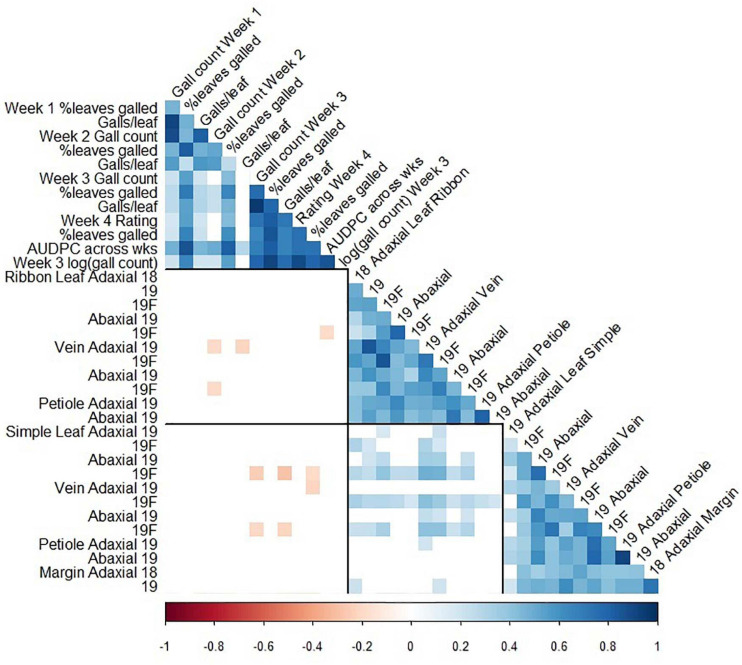
Pearson correlation coefficients between foliar phylloxera traits (Clark et al., 2018) and trichome density traits in GE1025, a cold-hardy hybrid grape population. Blank cells: non-significant correlation coefficient; black lines divide the matrix into phylloxera traits, ribbon trichome density traits, and simple trichome density traits; 18: 2018 forced dormant cuttings, 19: 2019 forced dormant cuttings, 19F: 2019 field-grown leaves.

The associations between trichomes and foliar phylloxera severity were non-significant except that trichomes on leaf blade and vein were significantly negatively correlated with phylloxera severity. The presence of simple trichome on leaf and vein, independent of the adaxial or abaxial surface, significantly affected the number of galls, percent leaves with galls, galls per leaf, visual rating, and area under the disease-progress curve (AUDPC; Supplementary Table 3). We found correlation coefficient (r) of -0.21 to-0.19 between ribbon density on vein with phylloxera severity at 2 weeks after infestation in one of two environments (Figure 5). We also found correlations (*r* = -0.28 to -0.19) between simple trichome density on vein and abaxial leaf with phylloxera severity at 3 and 4 weeks after infestation in one of the two environments (Figure 5). In addition to the weak phenotypic associations, QTL of trichome density and incidence was in different chromosomes from those found for phylloxera severity traits that were on chromosome 14 of the MN1264 map (Clark et al., 2018).

### Trichome QTL on Chromosome 1 Fine Mapped to 747 kb

The QTL from 11.7 to 20.7 Mbp (a 9 Mbp region) on chromosome 1 for ribbon trichome density on adaxial vein and adaxial leaf and from 10 to 20.7 Mbp (a 10.7 Mbp region) for simple density on abaxial vein was fine mapped to a 747 kb region from 12.63 to 13.38 Mbp on PN40024 v2 reference genome with three significant rhAmpSeq markers (Supplementary Figure 1 that also contains marker genetic and physical positions; for rhAmpSeq marker primer sequences, see Zou et al., 2020; Supplementary Dataset 1). Images for obtaining the phenotypic data are deposited at https://doi.org/10.13020/mqze-pw21. We found residual normality and a significant haplotype class effect of ANOVA for ribbon density on adaxial leaf, adaxial vein, and abaxial vein and simple density on adaxial vein and abaxial vein (Table 3). Individuals with L haplotypes at 12.63–13.38 Mbp were significantly lower than individuals with H haplotypes for ribbon density on adaxial leaf (square root transformed), ribbon density on adaxial vein, and simple density on abaxial vein (Table 3). For QTL on chromosome 10, we were not able to determine clear graphical genotypes segregating for ribbon trichome density after controlling for the chromosome 1 QTL (data not shown). For QTL on chromosome 1 for simple trichome density on adaxial vein, the graphical genotypes showed that the region 14.68–14.79 Mbp might be responsible, but Tukey’s HSD showed no difference of this trait between H and L haplotypes at this region (Table 3).

**TABLE 3:** Analysis of variance *p*-values for trichome density in October 2019 fitting a linear model of effects of haplotype (Hap), replication (Rep), rater (Rater), and number of genotypes within each haplotype (Hap:NoGeno).

Analysis	Effect	√adaLHDr	√adaVHDr	abaVHDr	log_e_adaVHDs	abaVHDs
ANOVA	Hap	<0.001	<0.001	<0.001	<0.001	<0.001
	Rep	<0.01	<0.01	<0.001	<0.01	ns
	Rater	<0.001	<0.001	<0.01	–	–
	Hap:NoGeno	<0.001	<0.001	<0.001	<0.001	<0.001
Tukey’s HSD	Mean difference μ_L_–μ_H_	-0.87	-1.22	1.12	0.04	-1.40
	*P*-value	0.0072	<0.001	ns (0.087)	ns	0.042

**Tukey’s HSD difference between means of low (L) and high trichome density (H) haplotypes at 12.6–13.4 Mbp on chromosome 1. ada, adaxial; aba, abaxial; LHD, leaf hair density; VHD, vein hair density; r, ribbon trichome; s, simple trichome; √, square root transformation; log_e_, natural log transformation. AbaLHDr, adaLHDs, and abaLHDs did not have normally distributed residuals after transformation.**

### Candidate Trichome Genes

#### Shotgun Sequencing at the Fine Mapped Region

**FIGURE 6:**
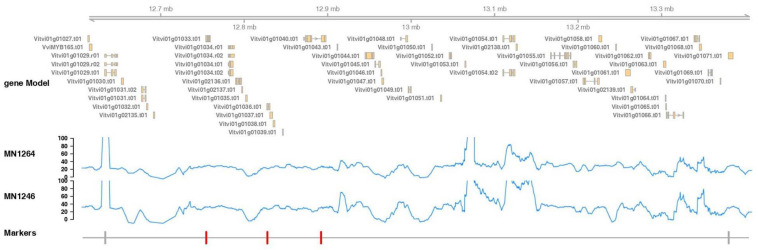
Marker positions, gene annotation, and reads coverage for the whole-genome shotgun sequencing of MN1264 and MN1246 at the fine mapped trichome density region, 12.6–13.4 Mbp on chromosome 1 (PN40024 12X v2, annotation v3). MN1264 and MN1246 are parents of the cold-hardy hybrid grape population. The gene annotation is illustrated in the upper panel. The read depth is plotted in the middle panel. Region with coverage over 100 × was ignored because it represents repetitive regions on the chromosome. The markers significantly associated with trichome density are in red, and the closest flanking markers are in gray.

The fine mapped region contained 45 candidate genes, of which 30 have annotations based on the PN40024 12X reference v3 annotation (CRIBI) (Figure 6 and Supplementary Table 4). The whole-genome shotgun coverage of MN1264 and MN1246 at the fine mapped region containing these genes is shown in Figure 6. Candidate genes encoding products related to trichome are expansin, pentatricopeptide repeat protein, ULP protease, NAC transcription factor 29, EF-hand protein, MYB140, O-fucosyltransferase family protein, and PCI protein. Of these candidate genes, MN1264 and MN1246 have a high impact variation (stop codon) at NAC transcription factor 29 and a moderate impact variation (missense) at EF-hand protein (Supplementary Table 5).

#### Nanopore Sequencing of Three Previously Reported Genes on Chromosome 1

The three candidate genes suggested by Barba et al. (2019) include transcription factor WEREWOLF (WER), zinc finger protein 5 (ZFP5), and SCARECROW-LIKE (SCR-LIKE) protein 21 (based on NCBI annotation; Barba et al., 2019, were based on CRIBI v2.1 annotation) at 8.1, 9.7, and 19.2 Mbp on the PN40024 12X reference. Bam files of the nanopore data are deposited at https://doi.org/10.13020/mqze-pw21. Sequence variations at candidate genes WER and SCR-LIKE 21 were obtained from nanopore sequencing, whereas variations at candidate gene ZFP5 were obtained from whole-genome shotgun sequencing because of the low read depth of nanopore sequencing at this gene.

The long-range PCR nanopore sequencing results show a polymorphism at the end of the exon of the first candidate gene (transcription factor WER) between the parents. MN1264 had an 8 bp homozygous deletion, and MN1246 had an 8 bp heterozygous deletion (Supplementary Figure 3). For the second candidate gene (ZFP5), there were several insertion–deletion differences between MN1264 and MN1246 upstream of ZFP5. For example, upstream of the exon of ZFP5, MN1264 had homozygous deletions at two sites where MN1246 had a heterozygous deletion or no variation; MN1246 had a 10 bp heterozygous deletion at a third site with no insertion–deletion polymorphism in MN1264 (data not shown). For the third candidate gene (SCR-LIKE 21), MN1264 had a 16 bp heterozygous insertion, and MN1246 had an 8 bp heterozygous deletion around this region at the beginning of the coding sequence (data not shown).

#### Comparison of Trichome Density on “Pinot” Mutants at SCR-LIKE 21/GAI1

Because SCR-LIKE 21 has high structural and sequence similarity to GIBBERELLIN-INSENSITIVE (GAI) in *Arabidopsis thaliana* (Pysh et al., 1999), we investigated how grape mutants at this potential candidate gene differed for trichome density. Ribbon trichome density on leaf blade was higher in “Pinot Meunier” than in “Pinot Noir,” whereas there was no difference between “Pinot Noir” and “Pinot Pixie” (almost significantly higher in “Pinot Pixie” on adaxial leaf) (Supplementary Figure 4A). There were no differences for simple trichome density among “Pinot Noir,” “Pinot Meunier,” and “Pinot Pixie” (Supplementary Figure 4B).

## Discussion

The QTL on chromosome 1 found in this study was previously identified by Barba et al. (2019), and we further fine mapped the QTL. They reported QTL on chromosome 1 from 8.7 to 15.5 Mbp for adaxial ribbon density and from 10.1 to 18.4 Mbp for simple density using a hybrid grape population of “Horizon” × Illinois 547-1. We validated their QTL in a different genetic background using a Minnesota hybrid grape population with genomic contributions from six *Vitis* species (Teh et al., 2017). Due to different populations studied and the different phenotyping methods used, our QTL (10–20.7 Mbp on chromosome 1) was slightly shifted but overlapping from that found by Barba et al. (2019). We further fine mapped the QTL for three traits: ribbon density on adaxial vein, ribbon density on adaxial leaf, and simple density on abaxial vein to a 140 kb region (12.75–12.89 Mbp) and sequenced the candidate genes within this region and surrounding this region on chromosome 1 suggested previously by Barba et al. (2019) in the parents of our mapping populations, MN1264 and MN1246.

The results were consistent across different years/experiments. The high correlations between trichome density on forced cuttings in 2 years and on field-grown leaves suggested repeatability of the experiment. Together with a relatively high heritability (*h*^2^ = 64–95%), trichome density is a trait with a limited environmental effect. In addition, the consistency of observing “Edelweiss” as having more ribbon trichomes than “Frontenac” across experiments confirmed these results.

Phenotyping resolution, such as scanner resolution, can be improved for simple trichomes on adaxial surfaces. Other populations with more trichome variation on the leaf blade shall be investigated, because the MN1264 × MN1246 populations had limited segregation across the population for trichome density on leaf blade, as seen by the non-normal residuals of the fitted linear models (Tables 1, 2). This likely led to QTL mapping difficulties and potentially contributed to the low correlation with phylloxera resistance. Alternative methods of trichome scoring should be investigated, such as scoring on multiple leaves as done by Barba et al. (2019), where we only scored a single leaf position. The fine mapping experiment was not replicated, and our results serve as a starting place for future validation studies. Even though the QTL mapping experiment was replicated, we saw relatively high variability within each genotype (Supplementary Figures 2, 4). It might be due to the variability in trichome scores between the last tender and first mature leaves. Future work should look at developmental regulation in trichome within a single genotype. Another possible limitation was the high SNP error rate of nanopore technology, limiting the analysis to only insertion, and deletion variants (Chaisson et al., 2019). We investigated whole-genome shotgun sequencing data on the parents. However, due to low coverage, we were only able to identify sequence variation at one candidate pseudogene within our fine mapped region.

Nanopore sequencing of the three candidate genes from Barba et al. (2019) was conducted before we obtained the fine mapping results, with the hope that we would recover some overlapping candidate genes. However, the candidate genes identified within our fine mapped region (12.6–13.4 Mbp on chromosome 1) were separate from these three candidate genes (8.5, 10.1, and 20.4 Mbp on chromosome 1) when compared using the same PN40024 12X v2 reference. Although outside of the fine mapped region, we observed potential functional variations at these three candidate genes. These three candidate genes, together with candidate genes in our fine mapped region, serve as a starting point for future studies as more relevant genome sequences become available that are related to the mapping populations. Our results suggest potential new trichome candidate genes in the cold-hardy hybrid grape materials separate from those already identified.

Within the fine mapped region, variations were detected in MN1264 and MN1246 including stop codons at candidate genes NAC transcription factor 29, MN1264 has one functional copy of NAC transcription factor 29 and the other copy with a stop codon, while MN1246 has both copies with a stop codon at this gene. NAC transcription factor 29, through a tissue-specific expression analysis using quantitative real-time PCR, was found to be specifically expressed in cucumber *Cucumis sativus* L. fruit trichomes and might have responsive elements to GA, ethylene, IAA, and/or methyl jasmonate (Liu et al., 2018). Variations of MN1264 and MN1246 also (Variations of MN1264 and MN1246 also) exist at the EF-hand protein, where MN1246 has a missense at both copies at this gene while MN1264 has one functional copy of this gene. One EF-hand Ca^2+^ binding protein was shown to play a role in cell branching in trichome (Dobney et al., 2009). Although no annotation was found, MYB140 is another potential trichome candidate gene. MYB genes have been reported to be important in trichome formation in *Arabidopsis* (GL1), *Antirrhinum majus*, *Nicotiana tabacum*, *Gossypium hirsutum*, and root hair formation in *Arabidopsis* (Wada et al., 1997; Payne et al., 1999).

The first candidate gene proposed by Barba et al. (2019), transcription factor WER, regulates a negative regulator in lipid production in *A. thaliana* GLABRA2 (GL2) and regulates hairless cell fate (summarized by Yao et al., 2013). The homozygous deletion observed in MN1264 at the end of transcription factor WER suggested loss of both functional gene copies and might explain why there was a dense trichome phenotype in MN1264 (ribbon trichomes on abaxial vein, adaxial leaf, and adaxial vein and simple trichomes on abaxial vein). It remains to be known if MN1069 loses both copies at the same genetic position. MN1246 had a heterozygous deletion at this position that can suggest MN1246 retains one functional copy of hairless allele explaining the less dense trichome phenotype, assuming that the gene had an additive effect. For the second candidate gene, ZFP5 is a C2H2 zinc finger protein that controls trichome cell development through GA and cytokinin signaling in *Arabidopsis* (Sun et al., 2015). The overexpression of ZFP5 results in ectopic trichome formation on carpels and other inflorescence organs, whereas the loss-of-function results in a reduced number of trichomes (Zhou et al., 2011). Whether the sequence variations upstream and in the introns of ZFP5 led to phenotype variation, we did not know. Sequence and expression analyses may reveal differential expressions at these regions between MN1264 and MN1246. Gene knock-out strategies as performed in other crops (Gao et al., 2013) can also be conducted on this and other candidate genes in MN1264 to validate gene functions. The third candidate gene, SCR and SCR-LIKE, is required for proper radial patterning of roots and shoots and has high structural and sequence similarity to GIBBERELLIN-INSENSITIVE (GAI) and RGA in *A. thaliana* (Pysh et al., 1999). DELLA GAI proteins are negative regulators of GA responses (Ariizumi et al., 2008). The insertion observed in MN1264 at the beginning of SCR-LIKE 21 suggested that MN1264 lost one functional copy of SCR-LIKE 21. In all, there are several candidate genes reported/suggested on chromosome 1, which are “hotspots” for future studies of trichome diversity on foliage and canes in cold-hardy grape and other native grape materials.

To confirm the role of SCR-LIKE 21/GAI1 gene in trichome density, we compared the trichome density of “Pinot Noir,” “Pinot Meunier,” and “Pinot Pixie.” “Pinot Meunier” has a mutation (GAI1/gai1) in the L1 layer of “Pinot Noir” (Skene and Barlass, 1983). “Pinot Pixie” is heterozygous GAI1/gai1 as “Pinot Meunier” but has the mutation throughout the whole plant (Skene and Barlass, 1983; Boss and Thomas, 2002). Our hypothesis was that “Pinot Noir” has lower trichome density than “Pinot Meunier” and “Pinot Pixie,” both of which have high trichome density because of the disruption in one allele of the GAI1 gene. This hypothesis was confirmed for ribbon type on leaf blade between “Pinot Noir” and “Pinot Meunier,” whereas there was less of a significant difference between “Pinot Noir” and “Pinot Pixie.” This result suggests that GAI1 not only keeps a brake on plant growth until activation by GA to ensure later rising structures become tendrils rather than flowing stems but also suppresses hair growth (Smyth, 2002). Thus, when GAI1 is disrupted, the plant shows dwarfness, continuous inflorescence, and dense trichomes (Smyth, 2002). This result agrees with that of Skene and Barlass (1983) that “Pinot Meunier” obtained the dense trichome phenotype from a mutation in the L1 layer of “Pinot Noir,” and that “Pinot Pixie” did not retain this dense phenotype suggesting that culture of apical fragments has led to the breakdown of genetic stability. However, it could also be that our method of scoring trichomes on a single leaf was not sufficient to capture the phenotype of the whole plant as did Skene and Barlass (1983).

Trichomes on leaf blade and vein, regardless of the side of leaf, perhaps impede phylloxera crawler movement. It makes sense as phylloxera is known to infest young leaf tissues and trichomes on young tissues perhaps play a role in impeding phylloxera gall development. One interesting observation was that ribbon trichomes seemed to impact phylloxera severity soon after the initial infestation (2 weeks) or throughout the period after infestation (1–4 weeks), whereas simple trichomes seemed to be important later after infestation (3–4 weeks). This suggested that ribbon trichome possibly served a more general role in plant defense, whereas simple trichomes served a more specific role. Furthermore, more trichome incidence traits seemed to be related to phylloxera severity. It could be that the QTL of trichome density was minor QTL for phylloxera resistance that was not detected in the relatively small population used (GE1025, *N* = ∼125). However, these hypotheses need further investigation. To be noted is that gall formation accompanies trichome formation at the opening of the gall (Sterling, 1952).

Trichome density itself is important for breeding purposes because of its association with disease resistance traits. QTL for trichome density has been found to co-localize with QTL for predatory mite and downy mildew resistance (Chitwood et al., 2014; Kono et al., 2018; Barba et al., 2019). Kono et al. (2018) found a major QTL on chromosome 5 for abaxial ribbon trichome density that was also a small effect QTL for downy mildew resistance in hybrid grape populations of *V. labrusca* origin. The adaxial ribbon and simple trichome density found by Barba et al. (2019) on chromosome 1 were co-localized with the QTL for predatory mite abundance; more trichomes were associated with higher predatory mite abundance. Thus, depending on the breeding objectives, trichome-dense phenotypes can be selected for (or against).

Trichome density is most prominently related to leaf shape, which plays an important role in grapevine taxonomy (Chitwood et al., 2014). Leaf shape confers adaptive advantages to the plant, such as temperature regulation, photosynthetic capacity, and water use (summarized by Demmings et al., 2019). Interestingly, some of these leaf shape traits including leaf circularity, petiolar junction to inferior branch points, and leaf lobing were also mapped to chromosome 1 (Chitwood et al., 2014). The QTL on chromosome 1 for leaf lobing was also detected using *V. vinifera* or hybrid populations (Welter et al., 2007; Demmings et al., 2019). Demmings et al. (2019) suggested candidate genes associated with leaf lobing, such as DELLA family GRAS transcription factor VviRGA5. If the relationship of leaf shape, trichome density, and disease and insect resistance can be established (tightly linked or pleiotropic), breeding for disease resistance can be accelerated through selection for leaf shape and trichome. Leaf shape traits are relatively easy to phenotype and do not rely on inoculation, infestation, etc. We used both manual scoring of scanned leaf samples (Klein et al., 2017) and labor-intensive scoring under the dissecting scope. This allowed for more time for trichome scoring without the worry of leaf deterioration. If scanning resolution of leaf samples can be improved and be coupled with an automated pipeline to quantify trichome density, the phenotyping process can be achieved objectively in a high-throughput manner. Kono et al. (2018) used ImageJ and LP_Mouzi plugin to measure ribbon trichome density. One challenge we had was differentiating between ribbon and simple trichomes and could not use this methodology.

Although the current work was initiated to examine the relationship between foliar phylloxera resistance and trichome incidence/density, we found a weak yet significant relationship between them and provided better genetic knowledge for trichome density itself, for its role in hybrid grape breeding. Given the nature of multiple *Vitis* species in the pedigrees of cold-hardy hybrid breeding materials, it was challenging to make sense of the sequence variations in MN1264 and MN1246 through alignment to a single reference genome at a time. Those genes found on both the *V. vinifera* reference and the *Vitis riparia* reference are of special interests. As we investigated the “Pinot” mutations in the GAI1 locus, similar future work should be done at other candidate genes. To expand upon the current work, the ancestors of MN1264 and MN1246 could be investigated to understand which *Vitis* species did the high trichome density haplotype descended from. The grandparents MN1069 and “Frontenac” that are frequently used in the breeding program are likely the sources of high trichome density. Within the *Vitis* germplasm, there is opportunity to explore and introduce desirable traits from species, such as *Vitits amurensis*, that exhibit diversity for trichome density, type, and color (Cheng et al., 2013). A better understanding of the evolutionary advantages of trichomes, such as its role in speciation and pathogen/pest defense, could allow more targeted breeding and germplasm improvement.

## Data Availability Statement

The original contributions presented in the study are included in the article/Supplementary Material, further inquiries can be directed to the corresponding author/s. Phenotypic data and sequencing data has been deposited into the Data Repository for University of Minnesota (DRUM) (https://conservancy.umn.edu/handle/11299/214951).

## Author Contributions

LY conducted the research and wrote the manuscript. MC supervised the research and edited the manuscript. AK generated GE1783 rhAmpSeq data and constructed GE1783 linkage map. CZ generated GE1783 rhAmpSeq data and conducted SNPeff analysis for whole-genome shotgun data. LC-D helped with rhAmpSeq data generation and provided insights in phenotyping methods and “Pinot” mutants screening. AU collected GE1025 cuttings in 2018 and 2019 and scanned “Pinot” mutant images. PA conducted nanopore sequencing on three genes. DV provided nanopore sequencing equipment and supervision. ET generated consensus fasta files for candidate genes. All authors contributed to the article and approved the submitted version.

## Conflict of Interest

The authors declare that the research was conducted in the absence of any commercial or financial relationships that could be construed as a potential conflict of interest.
