# Supplementary material for: Fine Mapping of Leaf Trichome Density Revealed a 747-kb Region on Chromosome 1 in Cold-Hardy Hybrid Wine Grape Populations
Source: Front Plant Sci. 2021 Mar 3;12:587640. doi: 10.3389/fpls.2021.587640 (PMC7965957; doi:10.3389/fpls.2021.587640)
Supplement: Supplementary file 1 [file Data_Sheet_1.docx]

**

Supplementary Figure 1. Graphical genotypes of 72 unique haplotype classes (Hap) composed of 183 individuals of GE1783 at the ribbon (R) and simple (S) trichome density QTL on chromosome 1. Marker name x_yyyyy means on x chromosome at yyyyy physical position. Trichome density measured in October 2019 at ada: adaxial side of the leaf, aba: abaxial side of the leaf. The boxed region: fine mapped genetic region. Using the genotypic coding of LepMap3, “11” and “21” (“1” in the second position) represent the high (H) trichome density haplotype descended from MN1264 based on QTL mapping results of GE1025.


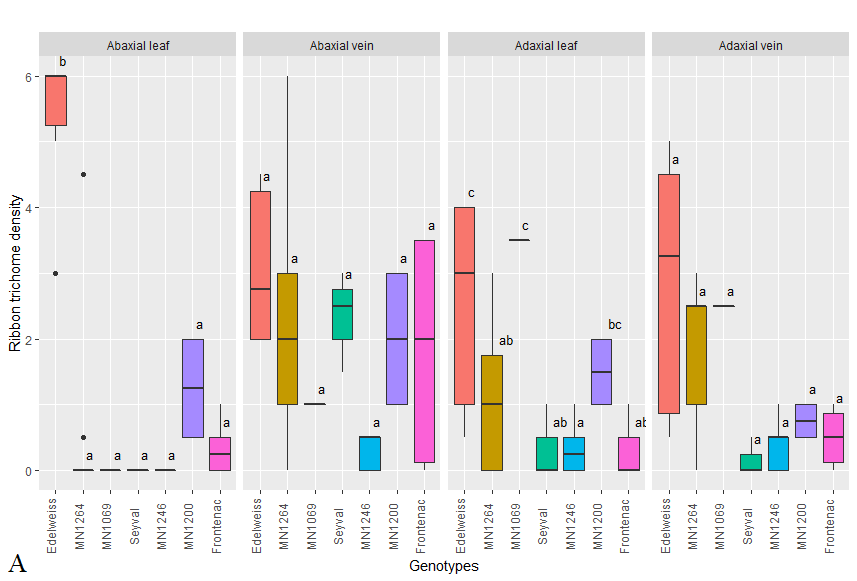


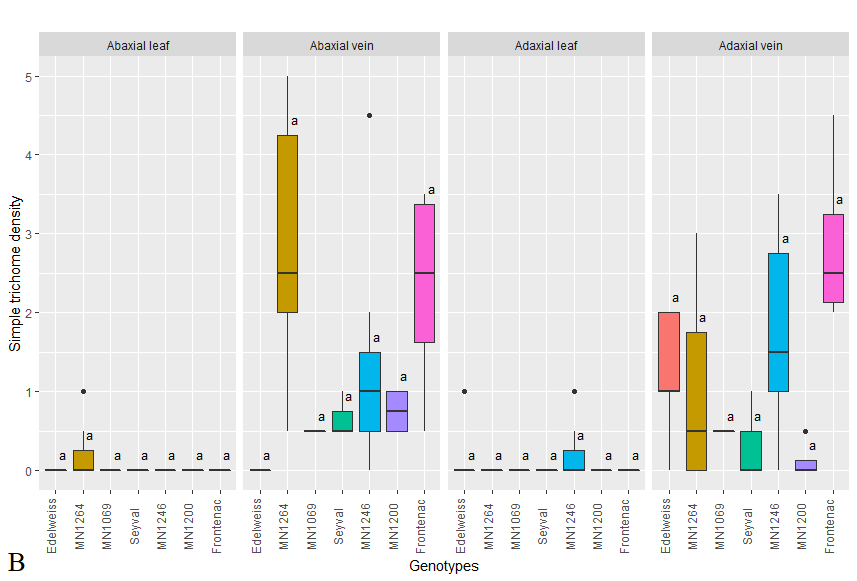


Supplementary Figure 2. **(A)** Ribbon and **(B)** simple trichome density on leaf and vein observed in ‘Edelweiss’, parents of GE1025/1783 (MN1264 and MN1246), and grandparents (MN1069, ‘Seyval Blanc’, MN1200, ‘Frontenac’). Letters indicate Tukey’s HSD differences among genotypes across environments (2018, 2019, 2019 field, and fine mapping experiment) within each leaf position.


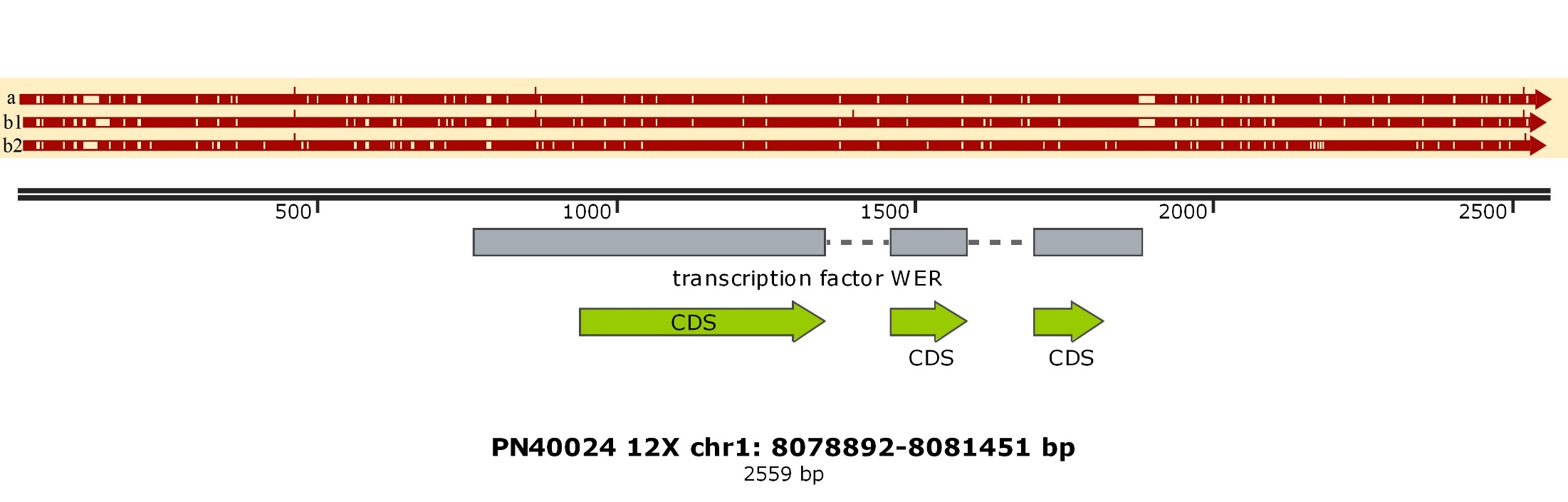


Supplementary Figure 3. Alignment of the first candidate gene sequences (nanopore) of (a) MN1264 and (b1 and b2) MN1246 from nanopore sequencing to PN40024 12X reference genome on chromosome 1. Red segment: sequence identical to the reference; white segment: deletion as compared to the reference; triangle: insertion as compared to the reference; gray box: exon; gray dash-line: intron; green arrow: coding sequence (CDS); yellow box: 5’ UTR.


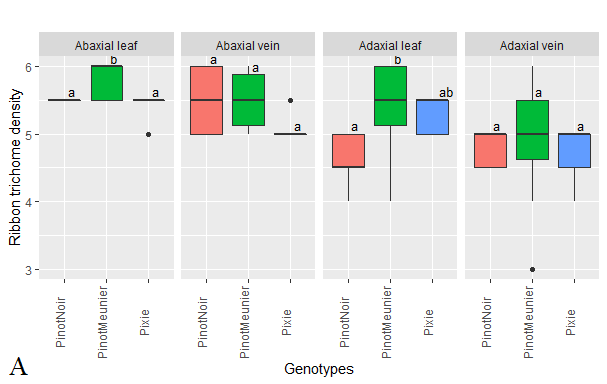


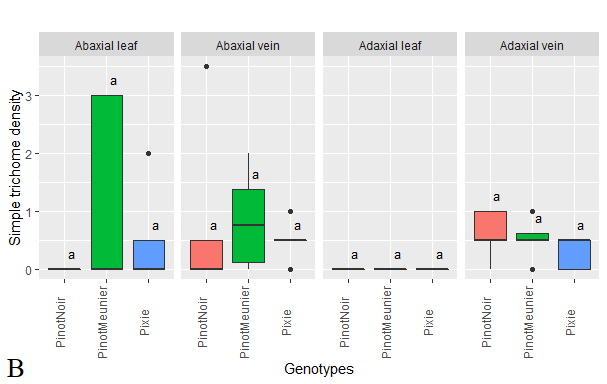


Supplementary Figure 4. **(A)** Ribbon and **(B)** simple trichome density measured in May 2020 of Pinot Noir, Pinot Meunier, and Pinot Pixie - mutants at candidate gene GIBBERELLIN-INSENSITIVE 1 (GAI1).
